# IL2RG, identified as overexpressed by RNA-seq profiling of pancreatic intraepithelial neoplasia, mediates pancreatic cancer growth

**DOI:** 10.18632/oncotarget.19848

**Published:** 2017-08-03

**Authors:** Michael Ayars, Eileen O’Sullivan, Anne Macgregor-Das, Koji Shindo, Haeryoung Kim, Michael Borges, Jun Yu, Ralph H. Hruban, Michael Goggins

**Affiliations:** ^1^ Department of Pathology, The Sol Goldman Pancreatic Cancer Research Center, The Johns Hopkins University School of Medicine, Baltimore, Maryland, USA; ^2^ Department of Oncology, The Sol Goldman Pancreatic Cancer Research Center, The Johns Hopkins University School of Medicine, Baltimore, Maryland, USA; ^3^ Department of Medicine, The Sol Goldman Pancreatic Cancer Research Center, The Johns Hopkins University School of Medicine, Baltimore, Maryland, USA

**Keywords:** pancreatic cancer, PanIN, RNA-seq, IL2RG, JAK3

## Abstract

Pancreatic ductal adenocarcinoma evolves from precursor lesions, the most common of which is pancreatic intraepithelial neoplasia (PanIN). We performed RNA-sequencing analysis of laser capture microdissected PanINs and normal pancreatic duct cells to identify differentially expressed genes between PanINs and normal pancreatic duct, and between low-grade and high-grade PanINs. One of the most highly overexpressed transcripts identified in PanIN is interleukin-2 receptor subunit gamma (*IL2RG*) encoding the common gamma chain, IL2Rγ. CRISPR-mediated knockout of *IL2RG* in orthotopically implanted pancreatic cancer cells resulted in attenuated tumor growth in mice and reduced JAK3 expression in orthotopic tumors. These results indicate that IL2Rγ/JAK3 signaling contributes to pancreatic cancer cell growth *in vivo*.

## INTRODUCTION

Pancreatic cancer is the third-leading cause of cancer death in the USA, with a 5-year survival rate of 8% [1]. Most pancreatic ductal adenocarcinomas are thought to arise from pancreatic intraepithelial neoplasia (PanIN) [2]. PanINs are small, microscopic lesions currently only identifiable by microscopic analysis of resected pancreata. PanINs have been classified according to their histological grade of dysplasia into low- (PanIN-1), intermediate- (PanIN-2) and high-grade lesions (PanIN-3) [3, 4]*.* A recent consensus conference recommended that PanINs should be classified as either low-grade or high-grade [4]. While low-grade PanINs are common in adults, few of these lesions ever progress to PanIN-3 [5, 6]. Most PanIN-3 lesions are found in association with an invasive ductal adenocarcinoma [2], but PanIN-3 lesions are also identified in the absence of cancer in pancreata of high-risk patients that have undergone pancreatic resection for concerning pancreatic imaging abnormalities during pancreatic screening [7, 8]. Although the major genetic alterations of PanINs have been identified [3, 9], few studies have characterized the transcriptional changes of PanINs [10]. Most of these studies have evaluated candidate genes identified as differential expressed in pancreatic cancer tissues. One study analyzed samples of PanINs containing its surrounding stroma [11]. Understanding the transcriptional alterations of PanIN cells requires isolating pure samples, but it is particularly challenging to obtain intact mRNA from PanINs given the high level of RNase activity in the pancreas [12]. An analysis of microdissected low-grade PanIN using cDNA microarrays has found differentially expression of extra-pancreatic foregut markers [10]. MicroRNA profiles of microdissected PanINs have also been undertaken and have identified numerous differentially expressed miRNAs, many of which are known to be differentially expressed in pancreatic cancers [13]. In this study, we performed RNAseq analysis of microdissected PanINs and normal pancreatic duct samples to identify differentially expressed transcripts. We chose to examine the function of one of the novel and most highly overexpressed transcripts in PanIN, *IL2RG,* a gene encoding the IL2 gamma receptor, Il2Rγ, which mediates proliferation signals through the JAK/Stat pathway.

## RESULTS AND DISCUSSION

### Differential gene expression in PanIN samples

**Figure 1:**
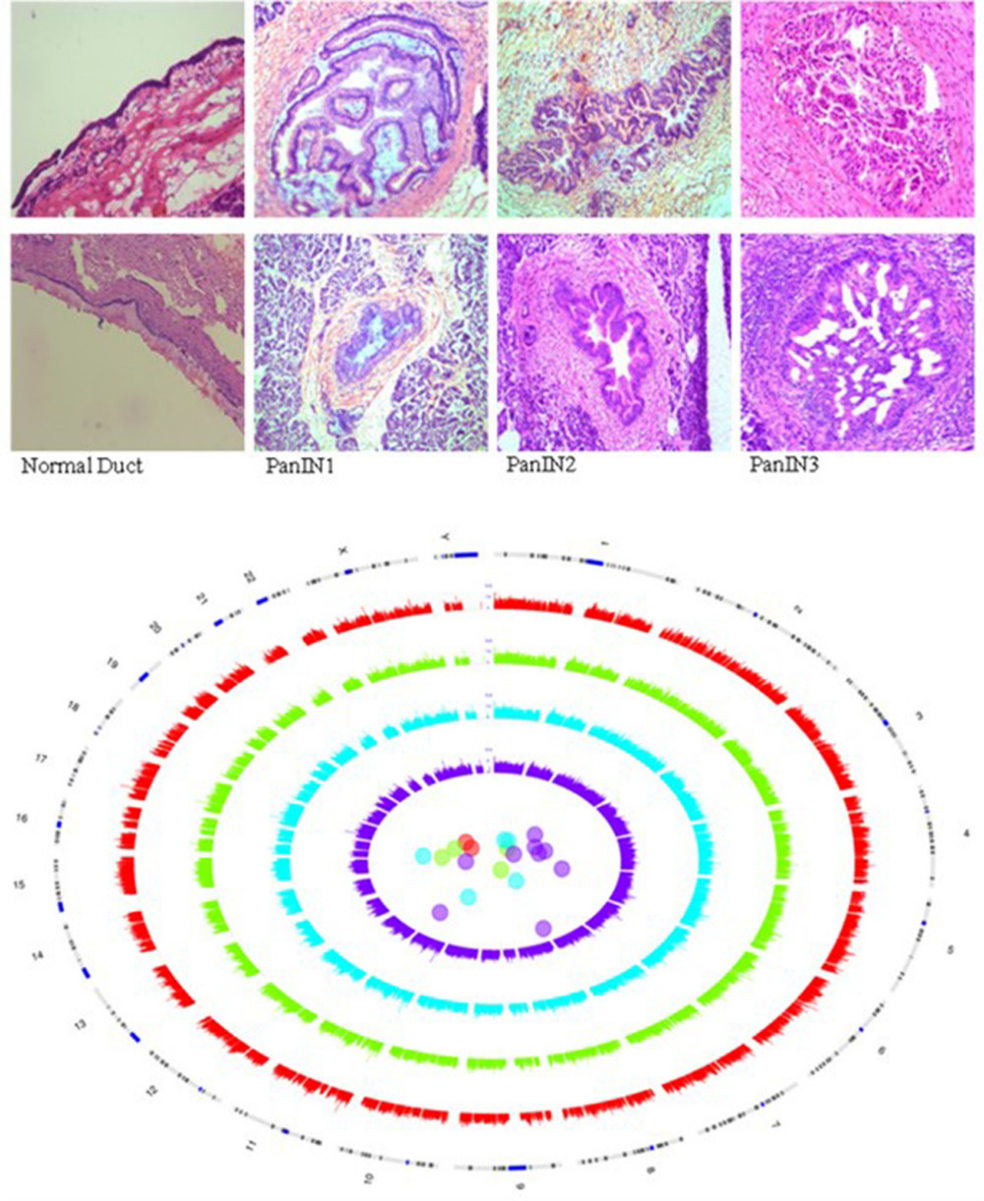
RNA-sequencing of laser microdissected PanINs Representative images of tissues in each sample group, H&E stained and photographed at 4x magnification. Circos and PCA plot of normal duct and PanIN samples. Red = normals, green = PanIN-1, blue = PanIN-2, purple = PanIN-3. Each grade of PanIN shows greater divergence in transcript expression between PanINs and from normal ducts than the last.

Twenty-one tissue samples were laser microdissected and processed for RNA-sequencing analysis: three normal pancreatic duct, four PanIN-1, five PanIN-2, and nine PanIN-3 samples. By principle component analysis, PanIN-3 samples were the most divergent PanINs from normal pancreatic duct samples (Figure 1). Table 1 lists some of the most significantly over- and underexpressed genes in PanIN samples reported to have functional roles in cancer. The full list of differentially (over- and underexpressed) expressed genes in PanIN vs. normal pancreatic duct samples determined using a q-value threshold including differential expression of alternatively spliced transcripts and neotranscripts is provided in Supplementary Tables. Supplementary Table S1 lists the total counts per million (TPM) for each transcript. Supplementary Table S2 lists differentially expressed genes between PanIN-1 vs. normal pancreatic duct samples. Supplementary Table S3 lists the differentially expressed genes between PanIN-3 vs. normal pancreatic duct and vs. all other samples. Supplementary Table S4 lists differentially expressed genes of all PanIN vs. normal pancreatic duct samples.

Table 1Differentially expressed genes in PanINs

**Table:** 

A	Gene name	ID	Log2 Fold Change	Adjusted p-value	B	Gene name	ID	Log2 Fold Change	Adjusted p-value
Regenerating Family Member 4		REG4	7.386	2.34E-02	Peptidase Inhibitor 3		PI3	4.5108	1.68E-02
FXYD domain containing ion transport regulator 3		FXYD3	5.0784	1.35E-04	Deleted In Lymphocytic Leukemia, 7		DLEU7	4.4313	1.10E-03
WAP four-disulfide core domain 2		WFDC2	4.7836	2.09E-02	Short Chain Dehydrogenase/Reductase Family 16C, Member 5		SDR16C5	3.2507	2.90E-03
annexin A10		ANXA10	4.5109	1.68E-02	Complement Component 4 Binding Protein Beta		C4BPB	3.0528	8.40E-03
interleukin 2 receptor subunit gamma		IL2RG	4.4313	1.11E-03	proteasome 26S subunit, ATPase 1 pseudogene 2		PSMC1P2	2.9991	2.90E-03
midkine (neurite growth-promoting factor 2)		MDK	4.2812	7.37E-05	Cadherin Related Family Member 2		CDHR2	2.982	2.40E-03
claudin 18		CLDN18	4.1737	1.37E-02	Chromosome 10 Open Reading Frame 10		C10orf10	2.5738	1.40E-02
thioredoxin interacting protein		TXNIP	4.1334	3.37E-03	Fer-1 Like Family Member 4, Pseudogene		FER1L4	2.512	2.09E-02
cathepsin E		CTSE	4.0083	4.98E-02	Leucine Rich Repeat Containing G Protein-Coupled Receptor 4		LGR4	2.2449	4.50E-03
peroxisome proliferator activated receptor gamma		PPARG	3.9705	2.11E-06	Cyclin G2		CCNG2	2.1469	2.18E-02
MDS1 and EVI1 complex locus		MECOM	3.767	3.75E-05	Steroid 5 Alpha-Reductase 1		SRD5A1	2.1433	1.10E-02
NAD(P)H quinone dehydrogenase 1		NQO1	2.4576	1.63E+03	Histone Cluster 1 H2A Family Member I		HIST1H2AI	2.1301	1.89E-02
isocitrate dehydrogenase (NADP(+)) 1, cytosolic		IDH1	2.4094	8.27E-03	RNA Exonuclease 2		REXO2	2.0702	1.25E-02
TIMP metallopeptidase inhibitor 1		TIMP1	1.5592	1.71E-02	Histone Cluster 2 H2A Family Member A3		HIST2H2AA3	2.0518	6.20E-03
cyclin dependent kinase inhibitor 1A		CDKN1A	-2.3596	3.32E-02	Coactosin Like F-Actin Binding Protein 1		COTL1	1.9507	1.00E-03
tissue factor pathway inhibitor 2		TFPI2	-3.6768	9.62E-06	SFT2 Domain Containing 2		SFT2D2	1.9217	4.50E-03
reelin		RELN	-3.9948	1.28E-04	Ribonucleotide Reductase Regulatory Subunit M2		RRM2	1.914	7.00E-04
secreted phosphoprotein 1		SPP1	-4.3801	2.91E-02	Family With Sequence Similarity 162 Member A		FAM162A	1.9028	2.04E-02
cystic fibrosis transmembrane conductance regulator		CFTR	-4.4648	6.01E-02	Phosphoglucomutase 2 Like 1		PGM2L1	1.882	1.74E-02

**Table:** 

C	Gene name	ID	log2 Fold Change	Adjusted p-value	D	Gene name	ID	log2 Fold Change	Adjusted p-value
lin-7 homolog A, crumbs cell polarity complex component		LIN7A	0.823	4.00E-04	vestigial like family member 1		VGLL1	-3.4168	1.38E-08
microtubule associated tumor suppressor candidate 2		MTUS2	0.7124	4.00E-04	A-kinase anchoring protein 7		AKAP7	-2.9597	1.38E-08
LY6/PLAUR domain containing 6B		LYPD6B	2.0132	4.00E-04	guanylate cyclase activator 2A		GUCA2A	-2.8986	1.38E-08
Leucine Rich Single-Pass Membrane Protein 2		C3orf45	1.285	4.00E-04	lin-7 homolog A, crumbs cell polarity complex component		LIN7A	-2.2845	1.38E-08
guanylate cyclase activator 2A		GUCA2A	1.0463	4.00E-04	gamma-aminobutyric acid type A receptor alpha5 subunit		GABRA5	-2.0514	1.38E-08
nitric oxide synthase trafficking		NOSTRIN	1.2269	4.00E-04	microtubule associated tumor suppressor candidate 2		MTUS2	-1.9404	1.38E-08
A-kinase anchoring protein 7		AKAP7	1.0568	4.00E-04	calneuron 1		CALN1	-1.7263	1.38E-08
vestigial like family member 1		VGLL1	1.1566	4.00E-04	coiled-coil domain containing 141		CCDC141	-1.5185	2.21E-08
STEAP4 metalloreductase		STEAP4	1.6482	4.00E-04	cannabinoid receptor 1		CNR1	-1.6342	5.05E-08
SRY-box 6		SOX6	0.9508	5.00E-04	leucine rich repeat containing 7		LRRC7	-1.8733	6.20E-08
dystrobrevin alpha		DTNA	0.9268	5.00E-04	solute carrier organic anion transporter family member 4C1		SLCO4C1	-2.5472	6.86E-08
coiled-coil domain containing 141		CCDC141	0.5392	8.00E-04	docking protein 5		DOK5	-3.3269	8.84E-08
calneuron 1		CALN1	0.5788	8.00E-04	POU class 6 homeobox 2		POU6F2	-3.2967	8.84E-08
cell adhesion molecule 1		CADM1	1.0328	8.00E-04	aldehyde dehydrogenase 1 family member A2		ALDH1A2	-2.6025	8.84E-08
gamma-aminobutyric acid type A receptor alpha5 subunit		GABRA5	0.6764	1.00E-03	transmembrane protein 27		TMEM27	-2.4447	8.84E-08
leucine rich repeat containing 7		LRRC7	0.6799	1.00E-03	STEAP4 metalloreductase		STEAP4	-3.8134	1.25E-07
solute carrier family 2 member 2		SLC2A2	2.3039	1.00E-03	MTUS2 antisense RNA 1		MTUS2-AS1	-2.0858	1.25E-07
solute carrier organic anion transporter family member 4C1		SLCO4C1	0.9252	1.10E-03	dystrobrevin alpha		DTNA	-2.1122	2.52E-07
neuropilin 1		NRP1	1.1469	1.60E-03	Leucine Rich Single-Pass Membrane Protein 2		C3orf45	-2.6853	3.05E-07
cannabinoid receptor 1		CNR1	0.5538	1.70E-03	apolipoprotein H		APOH	-1.7932	8.04E-07

A. Differentially expressed genes in PanIN-3 samples compared to normal pancreatic duct

B: Genes overexpressed only in PanIN-3 samples

C: Genes overexpressed in PanINs compared to normal pancreatic duct

D: Genes underexpressed in PanIN compared to normal pancreatic duct

**Figure 2:**
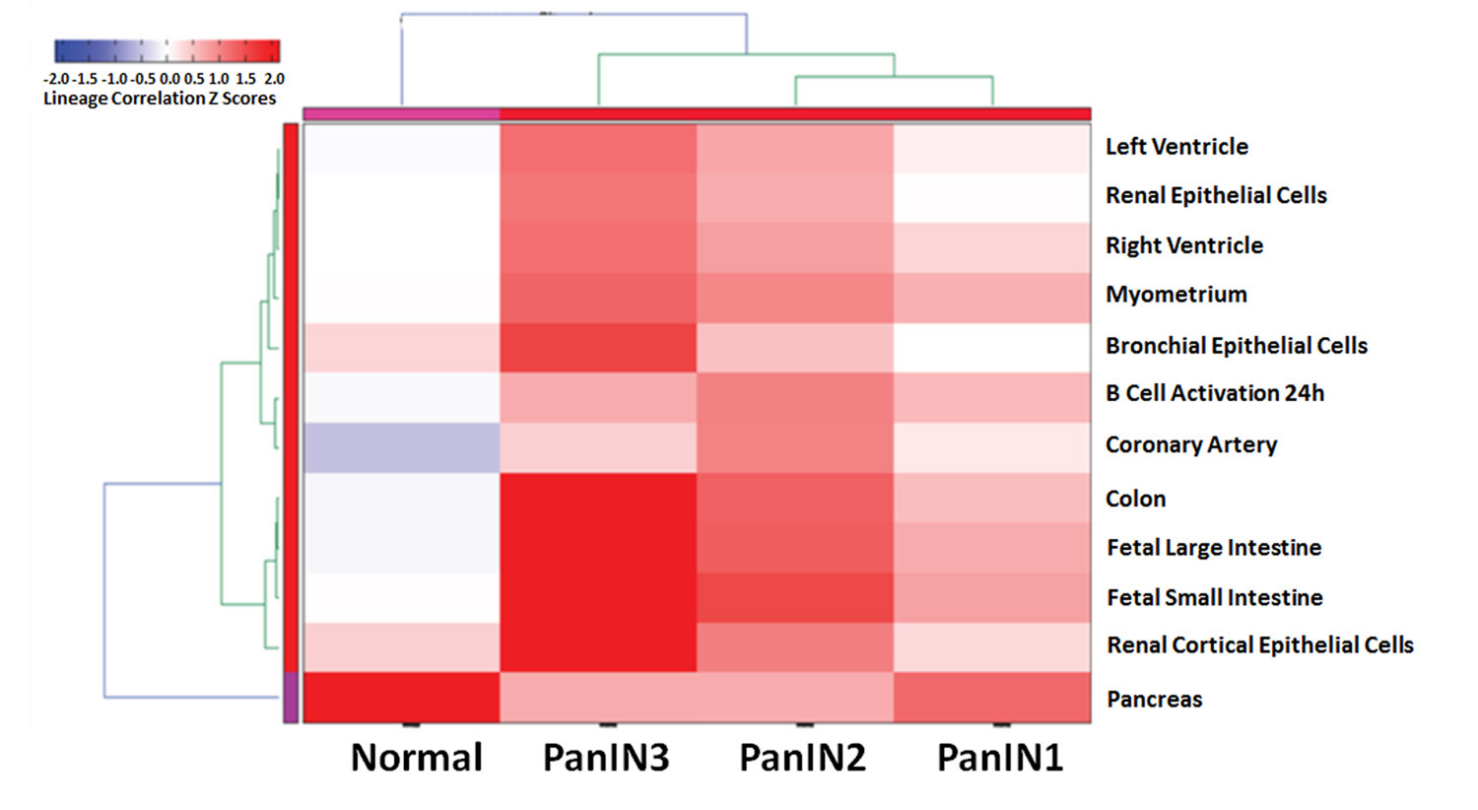
Global alternative splicing in PanINs Heirarchical heatmap clustering of similarity to tissue-based profiles of alternative splicing. Isoform expression in PanINs and normal ducts was compared to a curated database of isoform expression profiles in different tissues. The 12 profiles included in the heatmap are those that most strongly correlated with the PanIN and normal pancreatic duct samples. Normal pancreatic duct samples correlated strongly and almost exclusively with the normal pancreas profile. PanIN-2 and PanIN-3 samples were less related to the normal pancreas profile than PanIN-1 samples and more similar to other tissues (colon, fetal large intestine, and fetal small intestine).

We examined the lineage phenotype of PanIN cells using AltAnalyze. This tool compares isoform expression in our samples with a curated database of tissue lineages generated from the GEO public repository (https://sourceforge.net/p/altanalyze/wiki/LineageProfiler). The pattern of isoform expression in normal pancreatic duct samples correlated strongly with pancreas lineage and little else (Figure 2 and Supplementary Materials for further description). The isoforms expressed in PanIN-1 correlated most-strongly with the pancreas lineage whereas PanIN-2 and PanIN-3 isoform expression showed little correlation to the pancreas lineage, all PanINs also had strong correlations with the lineage profile of several other gastrointestinal tissues, including colon, fetal large intestine, and fetal small intestine.

**Figure 3:**
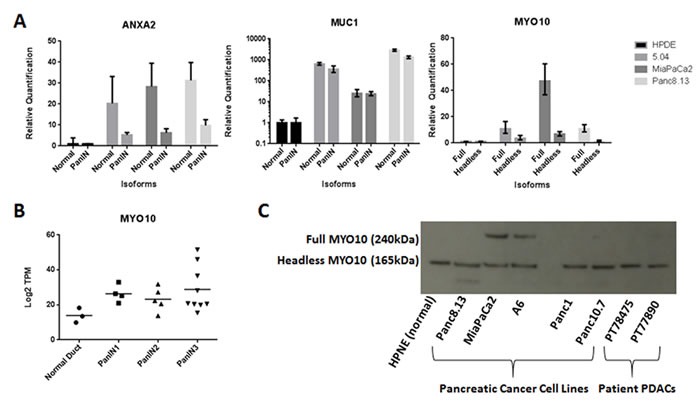
Alternative splicing in PanIN **A.** Relative quantification of isoform-specific mRNA in pancreatic cell lines. One normal pancreatic cell line (HPDE) and 3 cancer cell lines were assayed for specific isoforms of MUC1, ANXA2, and MYO10 by RT-PCR. Relative isoform mRNA levels after normalization to the corresponding 18S rRNA are shown. **B.** Total MYO10 mRNA expression is not significantly different in PanIN3 samples compared to normal pancreatic duct samples by RNA-seq. **C.** MYO10 protein isoform expression by Western blot in cell lines and patient samples. All tested samples are positive for the headless isoform (165 kDa). MiaPaCa2 and A6 pancreatic cancer cell lines are positive for the full isoform (240 kDa). HPNE = normal pancreatic cell line; Panc8.13, MiaPaCa2, A6, Panc1, and Panc10.7 = pancreatic cancer lines; PT78475 and PT77890 = primary pancreatic cancer samples.

Examples of differentially expressed alternative transcripts between PanIN and normal pancreatic duct samples included MUC1, ANXA2, and MYO10 (see Figure 3 and Supplementary Materials for further description). Expression of the full MYO10 isoform in developing motor neurons is associated with motility whereas the headless MYO10 isoform is a dominant negative inhibitor of full MYO10 [14]. Pancreatic cancer cell lines expressed both isoforms with higher levels of the full isoform (Figure 3).

Fusion transcript analysis was also performed using Top-hat fusion. Sixteen candidate fusion transcripts were identified each of which was detected in one PanIN sample (see Supplementary Table S5). For six of these events, one or both fusion partners has been previously reported in human cancer, although none of them have been reported in pancreatic cancer samples [15]. Many of the top 50 genes identified as overexpressed in PanIN-3 samples and listed in Table 1 have been reported as overexpressed in pancreatic cancer. Some significantly overexpressed genes of note include REG4, WFDC2, NQO1, UCP2, CLDN18, GATA4, IDH1, FXYD3 and CTSE. REG4 overexpression for example enhances the viability of pancreatic cancer cells and can be detected in the sera of patients with pancreatic cancer, although it is also expressed at lower levels in normal tissues and is not a specific circulating marker [16]. *WFDC2* (*HE4*), a well-known circulating marker of ovarian cancer, has been shown to be overexpressed in pancreatic cancers and elevated HE4 levels have been described in the sera of some patients with pancreatic cancer [17]. The redox gene *NQO1* is overexpressed in pancreatic and other cancers and is a promising therapeutic target [18]. Overexpression of the mitochondrial gene UCP2 helps uncouple mitochondrial oxidative phosphorylation and reduce oxidative stress. Prior studies have identified *CTSE* and *CLDN18* as overexpressed in pancreatic cancers and in PanINs [19, 20]. The overexpression of FXYD3 has been reported in both pancreatic cancer and PanINs, and FXYD3 has been reported to influence pancreatic cancer growth [21]. Some additional genes such as *TFF1, S100P* and *ANXA10* were identified as significantly overexpressed when all PanINs are compared to normal pancreatic duct samples. We were able to confirm the differential expression of 9 of the 49 differentially expressed genes previously identified in a cDNA microarray study by Prasad et al (in PanIN-1 vs normal pancreatic duct samples) [10] including TFF1, CTSE, ANXA10, S100P, SULT1C2 (overexpressed) and TFPI-2, CDKN1C (p57), SPP1, AGT (underexpressed).

**Figure 4:**
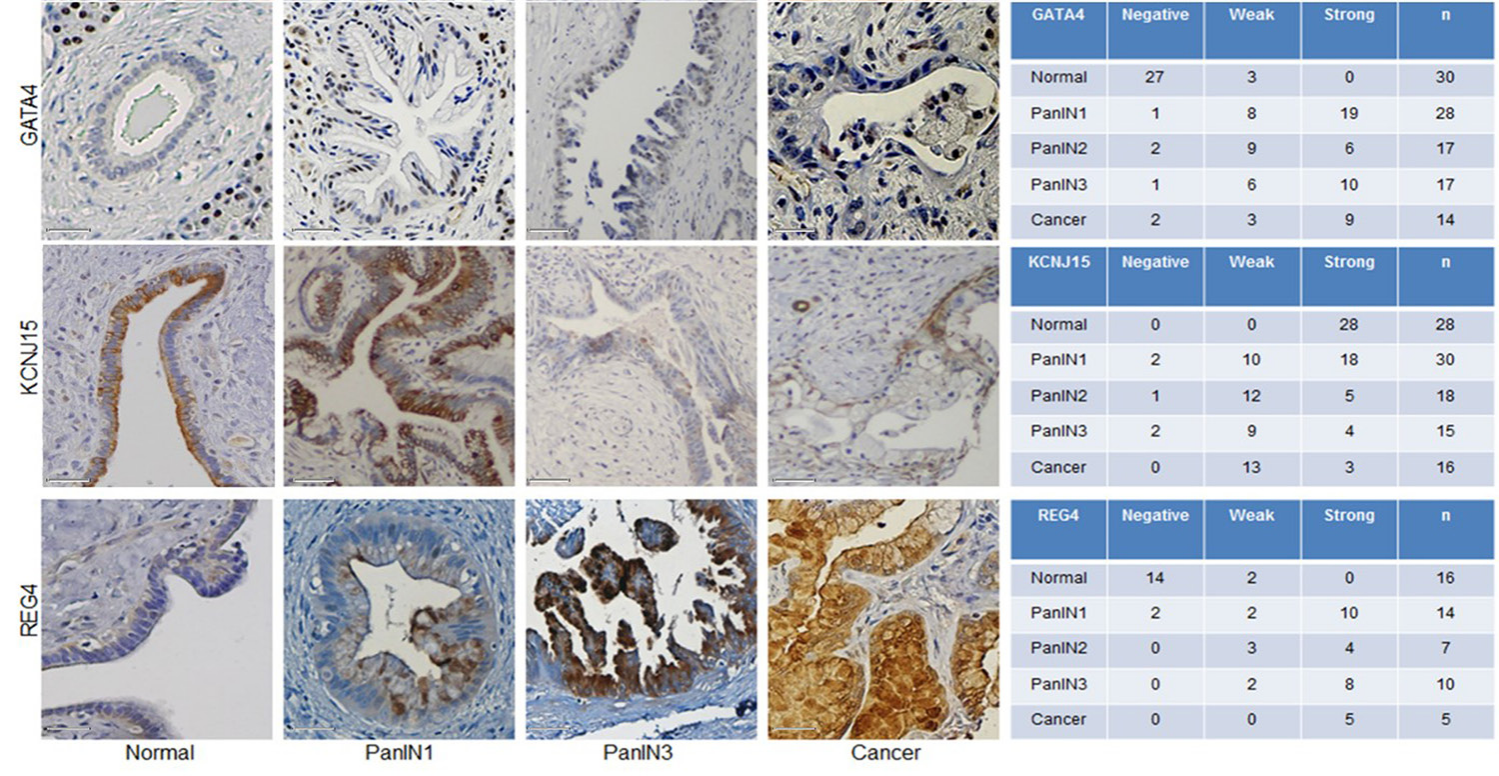
Protein expression alterations in PanIN and pancreatic adenocarcinoma Left, Representative immunohistochemistry images of normal pancreatic duct, PanINs and pancreatic cancer tissues for: Gata4, Kcnj15 and Reg4. Scale bar is 50 μm. Right, Number of tumor microarray cores at each labeling score (negative, weak, or strong).

**Figure 5:**
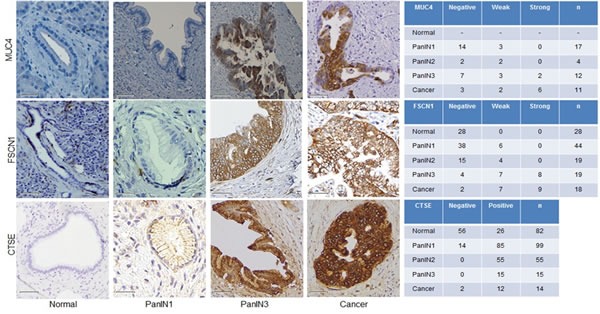
Expression of the protein products of transcripts identified as overexpressed in PanIN by RNA-seq Left, Immunohistochemistry (IHC) images representative of PanINs and pancreatic cancer tissues for three targets overexpressed by PanIN3s in our RNA-sequencing data: Muc4, Fscn1, and Ctse. Right, Number of tumor microarray cores at each labeling score (negative, weak, or strong) for MUC4, GATA4, and CTSE.

Reg4 was identified as overexpressed by immunohistochemistry in pancreatic cancer cells and PanINs with a progressive increase in expression with increasing PanIN grade (Figure 4). The loss of expression of the potassium channel KCNJ15 in PanIN samples was also confirmed by immunohistochemistry (Figure 4), which was consistent with the RNA-seq data which found high transcript levels in normal pancreatic duct samples and reduced expression in PanIN-3 samples (see Supplementary Table S3). FSCN1 transcript levels were minimal in normal pancreatic duct samples but were elevated in several PanIN-3 samples (Supplementary Table S3) and this pattern of expression was confirmed by immunohistochemistry, which found that ~half of all PanIN-3 samples and invasive adenocarcinomas had overexpression of FSCN1 (Figure 5). CTSE was one of the most highly differentially expressed transcripts in PanIN samples and immunohistochemistry confirmed overexpression in most PanIN and invasive adenocarcinoma samples compared to generally weak or absent expression in normal pancreatic ducts (Figure 5). Gata4, also among the most differentially expressed genes by RNA-seq, showed no expression in normal pancreatic duct samples by immunohistochemistry, although nuclear expression was noted in nearby acinar cells, and was overexpressed in most PanIN-3 and invasive pancreatic cancers (Figure 4). MUC4 overexpression in PanIN samples by immunohistochemistry was also evident (Figure 5). Several genes known to be silenced in pancreatic cancer were also found to be markedly underexpressed in the PanIN3 samples, including *TFPI2* and *RELN*, which are both frequent targets of silencing by DNA hypermethylation in pancreatic cancer and intraductal papillary mucinous neoplasms (IPMNs) [22, 23] [24]. One novel gene identified as significantly underexpressed in PanIN-3 samples was *CFTR. CFTR* has been reported to be underexpressed in pancreatitis tissues [25]. Loss of *CDKN1C* expression has been reported in pancreatic neoplasia [26].

### IL2RG expression

**Figure 6:**
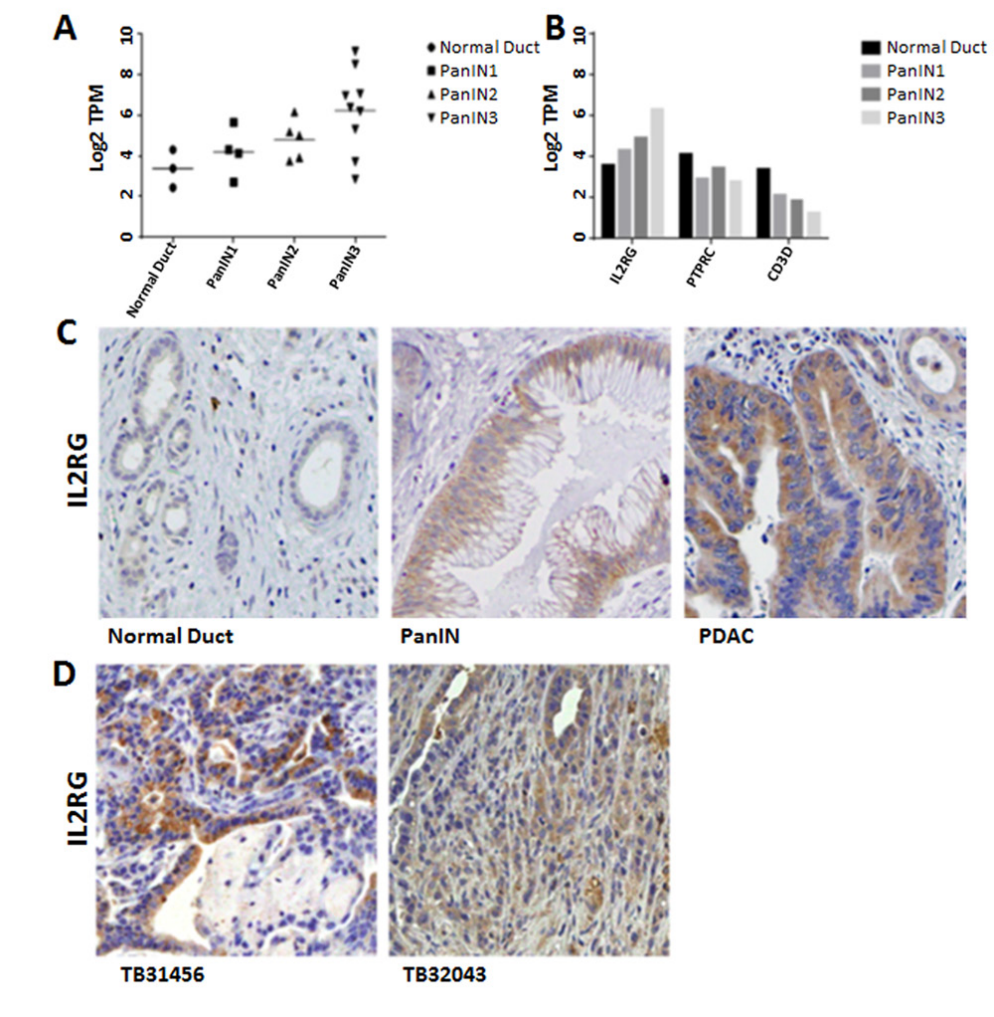
IL2RG overexpression in PanINs and pancreatic cancer **A.** Expression of IL2RG transcript in normal pancreatic duct and PanIN. Values are in Log_2_ transcripts per million (TPM). **B.** IL2RG and lymphocyte markers PTPRC and CD3D transcript levels in normal pancreatic duct and PanIN samples. **C.** Immunohistochemical analysis of IL2Rγ in human normal pancreatic duct, PanIN, and pancreatic cancer tissues, **D.** Orthotopic implants of mouse pancreatic cancer cell lines.

Many of the genes identified as significantly differentially expressed in PanINs have been reported to be differentially expressed in pancreatic ductal adenocarcinomas. One notable exception was *IL2RG* which was one of the most highly overexpressed genes in PanINs (average log2 fold change of 4.43 for PanIN-3 vs normal pancreatic duct, and the most differentially expressed gene among PanIN-3 vs. all other samples; Figure 6A). Lymphocyte markers such as CD45 (PTPRC) and CD3D were not enriched in PanIN samples (Figure 6B). The expression of IL2Rγ protein recapitulated RNA-seq results: immunohistochemical analysis of IL2Rγ protein in pancreatic tissues found little or no IL2Rγ in normal pancreatic duct cells but increasing expression with PanIN grade (Figure 6). Most primary pancreatic ductal adenocarcinomas had positive tumor immunolabeling of IL2Rγ (38/53 cases, 72%).

**Figure 7:**
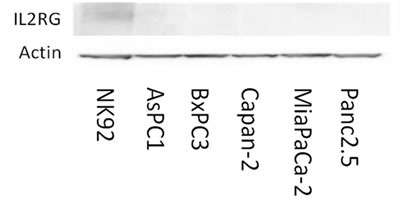
Pancreatic cancer cell lines do not express Il2rg protein *in vitro* NK-92, a natural killer cell line, is positive for Il2rg expression. Pancreatic cancer cell lines lacked detectable Il2rg expression.

IL2Rγ expression is rapidly lost *in vitro* [27] consistent with evidence that IL2Rγ undergoes rapid degradation (~1 hour) [28]. Consistent with this, we were unable to detect IL2Rγ protein in nine human pancreatic cancer cell lines by western blotting (Figure 7). IL2Rγ pathway activation was also not induced (no detectable Jak3 protein expression) by growing human pancreatic cancer cells as organoids (data not shown).

We hypothesized that induction of IL2Rγ expression in pancreatic cancer cells may require growing cells *in vivo* in the pancreas. We therefore examined the expression of IL2Rγ in five orthotopically implanted mouse pancreatic cancer cell lines by immunohistochemistry and identified expression of IL2Rγ in two of them (Figure 6C). These mouse cell lines did not have evidence of IL2Rγ pathway activation *in vitro* (no detectable JAK3 expression by western blot in either cell line).

### Generation of IL2RG knockout clones using CRISPR/Cas9

**Figure 8:**
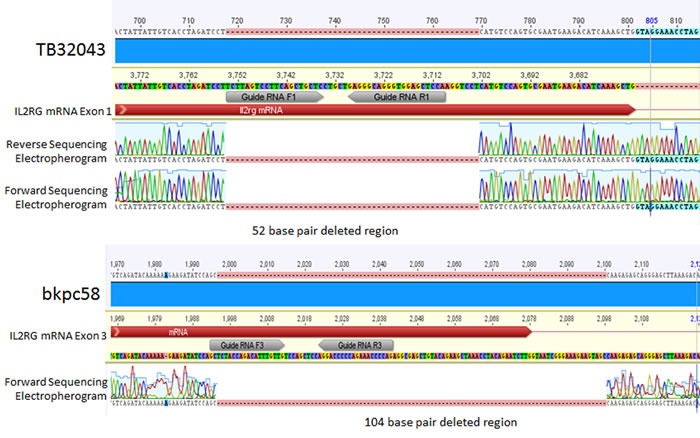
Electropherograms of IL2RG CRISPR-deleted regions in mouse cell lines Top, TB32043 is a female line with a biallelic 52 base pair deletion in exon 1 of IL2RG. Bottom, bkpc58 is a male line with a 104 base pair deletion in exon 3 of IL2RG.

To examine the effect of *IL2RG* knockout on *in vivo* growth we performed CRISPR/Cas9 knockout of *IL2RG* in mouse pancreatic cancer cell lines. Cells were transfected with plasmids containing the Cas9 nickase enzyme and pairs of guide RNAs targeting exons 1, 2, and 3 of *IL2RG*. Clones were isolated and tested for genome editing by Sanger sequencing. The two clones identified with genomic deletions in *IL2RG* were a 32043 clone with a 52 base pair deletion in exon 1, and a bkpc58 clone with a 104 base pair deletion in exon 3 (see Figure 8).

Attempts to induce the IL2Rγ pathway *in vitro* were not successful: The expression of IL2Rγ and proliferative responses to IL2Rγ ligands were not significantly affected by growth with cytokine-containing conditioned media from L929 cells either in the wild-type or IL2RGγ knockout cell lines. Similarly, the IL2Rγ ligands IL-4, GM-CSF or IL-7 did not significantly affect proliferation of either parental cells or *IL2RG* knockout cells.

### Impact of IL2RG knockout on the growth of orthotopically implanted pancreatic cancer cells

**Figure 9:**
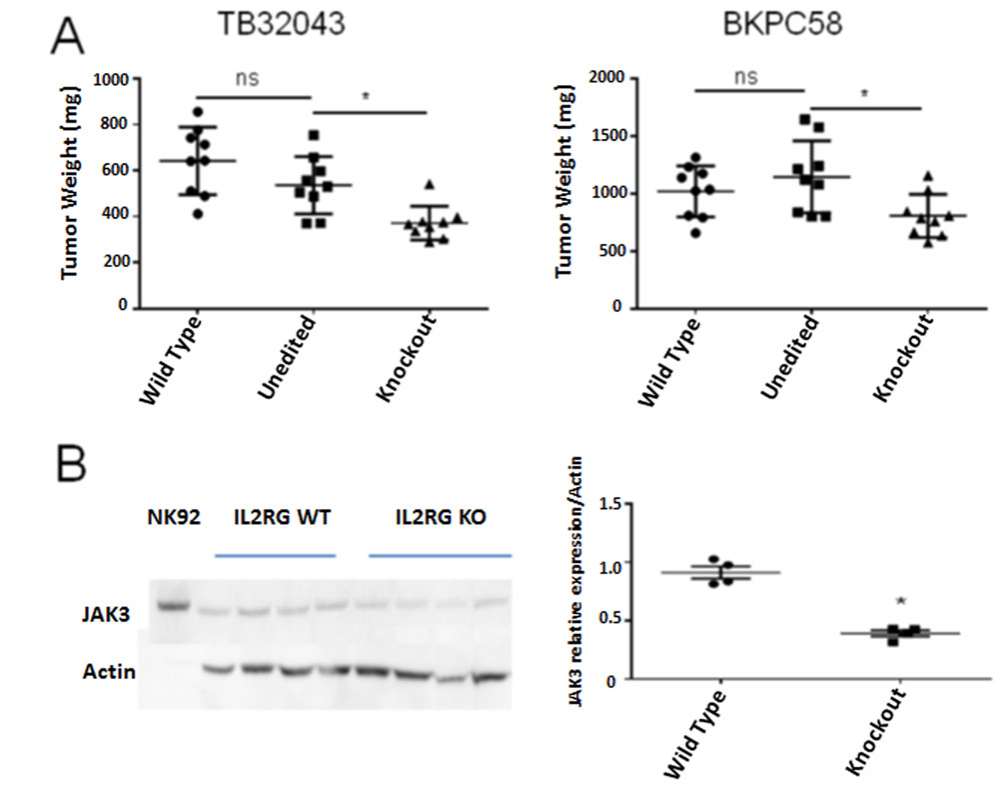
Reduced growth and JAK3 expression of pancreatic cancer cells with IL2RG knockout **A.** Tumor weights (mg) of orthotopically implanted TB32043 and BKPC58 cells. Tumors from *IL2RG* knockout cells were significantly smaller: TB32043 ∆30.75% ± 10.43% (*p*-value = 0.0001), bkpc58 ∆29.58% ± 12.58% (*p*-value = 0.0133). **B.** Western blot of JAK3 expression in dissected TB32043 xenograft tumors. Wild type tumors have significantly higher JAK3 expression than IL2RG knockout tumors (*p*-value 0.0156). Unedited cells are from clones that underwent the CRISPR editing but retained intact *IL2RG*.

We compared the growth of parental pancreatic cancer cells and cells with *IL2RG* knockout *in vitro* and found no significant difference (data not shown). We next injected CRISPR *IL2RG* knockout cells into mice to determine what effect *IL2RG* loss would have on *in vivo* tumor growth. After a sufficient period of *in vivo* growth (21 days for 32043 cells and 23 days for bkpc58 cells), injected mice were sacrificed and tumor weights compared. There was no significant difference in the weight of tumors generated from *IL2RG*-wild-type pancreatic cancer cells compared to CRISPR-unedited cells but there was a significant reduction in the weight of tumors generated from *IL2RG* knockout cells: TB32043 30.75% ± 10.43% (p = 0.0001), bkpc58 29.58% ± 12.58% (p = 0.0133) (Figure 9A). Additionally, western blot analysis of protein extracts from 32043 orthotopic tumors showed significantly higher JAK3 expression in parental vs. knockout tumors (p = 0.0156) (Figure 9B) consistent with reduced IL2Rγ/JAK3 pathway activation in the IL2RG-knockout pancreatic cancer cells.

In this study, we compare the transcript profiles of PanIN and normal pancreatic duct samples and identify differentially expressed transcripts. One novel gene we identify as overexpressed in PanINs and pancreatic ductal adenocarcinomas is the common gamma chain, IL2Rγ. CRISPR-induced truncating mutations of *IL2RG* in two pancreatic cancer cell lines resulted in significantly reduced cancer growth in orthotopic xenografts and reduced expression of its signaling partner JAK3, indicate that IL2Rγ signaling promotes proliferation of pancreatic cancer cells. It is likely that unique characteristics of IL2Rγ regulation, i.e. rapid loss of IL2Rγ expression in the absence of pathway activation, have precluded it from being previously identified as overexpressed in pancreatic cancer cells. IL2RG transcript expression detected in bulk primary pancreatic cancer samples could easily be attributed to the presence of lymphocytes in the tumor microenvironment. The rapid loss of expression of IL2Rγ in cells grown *in vitro* has limited our understanding of the IL2Rγ signaling pathway [29]. One candidate ligand and co-receptor that may signal through IL2Rγ in primary pancreatic cancer cells is granulocyte/macrophage colony stimulating factor (GMCSF) and its receptor GM-CSFRβ. Hematopoietic CD34^+^ cells responding to GM-CSF express GM-CSFRβ and IL2Rγ [30]. GMCSF is expressed in PanIN lesions [31] and promotes the growth, invasion, and metastatic potential of pancreatic cancer cells [32]. Other cytokines such as IL17 influence PanIN growth [33], but not through IL2Rγ. Our results also raise the possibility that targeting the IL2Rγ pathway such as with JAK3 inhibitors could limit the growth of IL2Rγ-expressing pancreatic cancers. JAK3 inhibitors are in clinical use to treat rheumatoid arthritis [34]. This could be tested in genetic engineered mouse models such as the KPC model where *in vivo* IL2Rγ overexpression would be expected to be retained in PanINs and pancreatic cancers. RNAseq profiles of PanINs and normal pancreatic duct can help investigators develop new hypotheses and evaluate candidate biomarkers of pancreatic neoplasia. Pancreatic imaging tests often identify subtle focal pancreatic abnormalities that turn out to be areas of focal PanINs [35]. RNA signatures of PanINs may have applications as diagnostic tests to identify PanINs *in situ*.

In conclusion, we have characterized the transcripts of PanINs and normal pancreatic duct samples using RNA-seq. Among the differentially expressed genes identified, *IL2RG* overexpression, identified as one of the most highly differentially expressed genes in PanINs, contributes to the growth of pancreatic cancer cells *in vivo*.

## MATERIALS AND METHODS

### Laser microdissection

The PanINs were obtained from the resection specimens of 10 patients with pancreatic ductal adenocarcinoma, two with intraductal papillary mucinous neoplasm (IPMN), one with serous cystadenoma, one with pancreatic neuroendocrine tumor, and one with chronic pancreatitis. Normal pancreatic duct samples were obtained from patients who had undergone pancreatic resection for low-grade IPMN or Pancreatic neuroendocrine tumor. PanINs identified on frozen sections were sectioned at 10 μm thickness carefully and quickly to preserve RNA. PanIN samples consisted only of dissections from a single PanIN; dissections were not performed on PanIN from different ductules even if they were on the same section as they could represent different PanIN lesions. Several thousand cells were dissected to have ~10 ug or more of mRNA for RNA-seq analysis. Tissue sections were mounted on activated polyethylene naphthalate slides, immersed in RNAlater (ThermoFisher Scientific) and stored at -80°C. Separate 4 μm sections were H/E stained for histological grading (performed by RHH, an experienced pancreatic pathologist). Slides were stained with Cresyl Violet and laser microdissected on a Leica LMD6 within 20 minutes of thawing. RNA was harvested from microdissected cells (Arcturus PicoPure RNA Isolation Kit) and concentration and quality measured using an Agilent Bioanalyzer with RNA 6000 Pico reagents. Only samples with an RNA integrity score (RIN) score of >7 were sequenced.

### RNA-sequencing

Thirteen of the harvested samples (one normal pancreatic duct, two PanIN-1, three PanIN-2, and seven PanIN-3 samples) were converted to cDNA libraries using a Nugen Ovation RNA-Seq V2 kit with the SOLiD fragment protocol and sequenced on an Applied Biosystems SOLiD v. 5500 Wildfire. These reads were aligned using Bioscope v1.3 Whole Transcriptome Plugin. FPKM values were calculated using HTSeq-count (http://www-huber.embl.de/HTSeq/doc/overview.html). The remaining eight samples (two normal pancreatic duct, two PanIN-1, two PanIN-2, and two PanIN-3 samples) were converted to cDNA libraries (Nugen kit) with the TruSeq protocol and sequenced on an Illumina HiSeq2500. Alignments and FPKM values were generated using rsem-1.2.9 (http://deweylab.github.io/RSEM/). FPKM values from the two sets of samples were combined using ComBat for batch removal. Lists of differentially expressed genes between the different grades of PanINs and normal pancreatic duct were generated using limma with an empirical Bayes method and fitted intensity trend.

Alternative transcripts were identified using the program AltAnalyze. AltAnalyze ( www.altanalyze.org) was run on BED files for RNA-sequencing data from 12 samples: three normal pancreatic duct and nine PanIN-3 samples. The EnsMart65 database was used as a reference. The ASPIRE algorithm, described in detail in the AltAnalyze manual ( http://www.altanalyze.org/help_main.htm), was used to score exon inclusion/exclusion events. In brief, a ratio is calculated separately for the inclusion and exclusion of a reciprocal junction, in which expression of the junction is divided by the mean of all gene expression reporting junctions and exons. The experimental group (PanIN-3) vs. control (normal pancreatic duct) ratios were then calculated, along with a false-discovery rate *p*-value (Benjamin-Hochberg correction). Lineage Analysis was performed by calculating correlation coefficients of the sample groups to Lineage WikiPathways networks.

Fusion detection analysis was performed on the eight samples (two normal pancreatic duct samples, and six PanIN samples, two of each PanIN grade) sequenced on the Illumina platform due to their higher read length. BED files were analyzed with Tophat-fusion ( http://ccb.jhu.edu/software/tophat/fusion_index.shtml). The fusion minimum distance was set to 100000000 and anchor length to 13. Tophat-fusion-post results were further filtered using Oncofuse (www.unav.es/genetica/oncofuse.html). *Fusion events were filtered out* if a fusion event was reported in a normal pancreatic duct sample, if the fusion event included a gene and partnered pseudogene, or if fusion partners were not in parallel or a coding orientation.

### Immunohistochemistry

Tissue microarrays (TMAs) of pancreatic ductal adenocarcinoma, PanIN and normal pancreas tissue from patients who had their pancreatic cancer resected at Johns Hopkins Hospital (Baltimore, MD) were analyzed as previously described [36].

The HRP EnVision^+^ System (DAKO Corp.) was used to evaluate IL2Rγ protein expression in TMAs using rabbit anti-IL2Rγ antibody (Sigma-Aldrich; 1:250 dilution). Expression of IL2Rγ in PanIN and cancer cells, when present was diffuse and uniform relative to its absence in normal pancreatic duct cells. Expression in lymphocytes was used as a positive internal control. Expression of mouse IL2Rγ was determined on tissue sections of orthotopic tumors generated from five mouse pancreatic cancer cell lines (a gift of Dr. Christine Iacobuzio-Donahue at MSKCC). Other antibodies used in immunohistochemical analyses included: Fscn1 (sc-56531, 1:100 dilution), Gata4 (sc-25310, 1:100 dilution), and Muc4 (sc-53945, 1:100 dilution) from Santa Cruz Biotechnology (CA, USA); Kcnj15 (NBP1-83091, 1:100 dilution) from Novus Biologicals (CA, USA); and Reg4 (AF1379) from R&D Systems (MN, USA). For Kcnj15, Gata4, Reg4, Muc4, and Fscn1, TMA cores were scored based on percentage of normal pancreatic duct, or neoplastic pancreatic expressing cells: negative (0% of cells), weak (0-50% of cells), or strong (50-100% of cells). Immunolabeling of expressing normal pancreatic duct and neoplastic pancreatic cells for Il2Ry and Ctse was diffuse and uniform. TMA cores were scored based on intensity for Il2Ry (negative, weak, or strong) and CTSE (negative or positive).

### Organoid culture

10,000 cells were pelleted at 4° C and resuspended in 4° C matrigel. 50 ul of matrigel were pipetted into the center of wells on a 24-well plate and placed in a 37° C incubator for 15 minutes to solidify. 500 ul DMEM + 10% FBS + 1% pen/strep was added to each well. Cells were passaged by aspirating media, adding ice-cold media to dissolve matrigel, and pipetting well contents into a 15-ml tube, pelleting, washing once with PBS, and resuspending cells in 4° C matrigel at a 1:2 dilution to seed additional wells.

### Cell culture

Mouse pancreatic cancer cell lines TB31456 and TB32043 were generously provided by Dr. Tuveson (Cold Spring Harbor Laboratory). bkpc58 was generated by Dr. Macgregor-Das while in Dr. Iacobuzio-Donahue's lab. These cell lines were derived from KPC mice (Kras G12D, P53 R172H, C57Bl6 background.

### Western blotting

Total protein lysates were extracted in RIPA buffer (Roche Diagnostics, Indianapolis, IN) with cOmplete Mini tablets (Roche) and homogenized (Diagenode Bioruptor, Denville, NJ) for 8 cycles (30s high, 30S off). Membranes were incubated overnight at 4° C with primary antibodies: rabbit anti-IL2Rγ (Santa Cruz Biotech., Dallas, TX), rabbit anti-JAK3 (Cell Signaling Technology, Danvers, MA), or goat anti-Actin (Santa Cruz), then incubated with horseradish peroxidase (HRP)-conjugated secondary antibody in 5% dry milk for 1 hour. Bound antibody was detected with a Pierce ECL Plus kit (ThermoScientific).

### Generation of CRISPR plasmids

CRISPR knockout experiments were performed with Cas9 nickase (Cas9n) enzymes guided by paired sgRNA sequences to mitigate off-target effects as previously described [37]. Paired sgRNA sequences targeting IL2RG on the X-chromosome were designed using the MIT CRISPR Design Tool ( crispr.mit.edu) and synthesized by Integrated DNA Technologies: Pair 1: 5’-TCTTAGTCCTTCAGCTGCTC-3’, 5’-GAGGGCAGGGTGGAGCTCCA-3’. Pair 2: 5’-TCCAGAGGTTCAGTGCTTTG-3’, 5’-TAGAGTACATGAATTGCACT-3’. SgRNA oligonucleotides were annealed and ligated into digested pSpCas9n(BB)-2A-GFP (PX461) (Addgene, Cambridge, MA) in 1 μl px461 (100 ng), 2 ul 10x Fast-Digest buffer, 1 μl each of annealed oligonucleotides (0.5 μM), Bbs1 Fast-Digest, T4 Ligase (all ThermoFisher Scientific), 14 μl ddH20 and incubated in a 37°C water bath for 2 hours and then transformed into Stbl3 bacteria by heat shock (ThermoFisher Scientific). Bacteria were selected ampicillin resistance (100 μg/ml) and harvested for plasmid (QIAprep Plasmid Miniprep kit Qiagen, Germantown, MD). Harvested plasmid was Sanger sequenced to confirm appropriate insertion.

### Generation of CRISPR knockout clones

Cells were plated on 24-well plates at 1.3×10^5^ cells/well 16-24 hours before transfection. Each well was transfected with 250 ng of each paired Cas9n plasmid using Lipofectamine 2000 (ThermoScientific Fisher) according to the manufacturer's protocol. The TB32043 cell line was transfected with the IL2RG-targeting pair one Cas9n plasmids and the bkpc58 cell line was transfected with the IL2RG-targeting pair two Cas9n plasmids. Three days after transfection, cells were flow-sorted for GFP expression into 96-well plates at one cell per well using a BSL2 FACSAria II instrument. Cells were grown for 3 weeks; their DNA then extracted to test for CRISPR deletions. Genomic DNA from the TB32043 cells was cloned into TOPO vectors for Sanger sequencing using TOPO TA Cloning Kit for Sequencing (ThermoScientific Fisher).

### Orthotopic implants of parental and CRISPR-knockout cells

3×10^5^ TB32043 cells or 1×10^5^ bkpc58 cells were injected into the pancreas of 6-10 week old C57BL/6J mice (The Jackson Laboratory, Bar Harbor, ME). Mice were sutured and followed daily for survival. After ~3 weeks, mice were sacrificed and dissected, tumors were weighed, immersed in RIPA buffer, and homogenized with a tissue disruptor to isolate protein for western blotting.

### *In vitro* proliferation

Pancreatic cancer cells were seeded in 60 wells of 96-well plates at 3,000 cells/well. After 24 hours, media was replaced with standard media, L929-conditioned media, or standard media supplemented with 100 ng/ul of an IL2Rγ ligand: IL4, GM-CSF, or IL7. After 72 hours, AlamarBlue was added to each well and cells incubated for an additional 4 hours. Absorbance at 490 nm was measured using a BMG FluoStar Galaxy instrument.

### Statistical analysis

Descriptive statistical values and plots were generated using the Microsoft Excel software packages, Graphpad Prism 6.0, and R bioconductor. Presented lists of genes were filtered to those differentially expressed with a q-value of < 0.1.

## SUPPLEMENTARY MATERIALS FIGURES AND TABLES













## Abbreviations

PanIN: pancreatic intraepithelial neoplasia
Il2Rγ: IL2 gamma receptor
IL: interleukin
GMCSF: granulocyte/macrophage colony stimulating factor
