# Supplementary material for: IL2RG, identified as overexpressed by RNA-seq profiling of pancreatic intraepithelial neoplasia, mediates pancreatic cancer growth
Source: Oncotarget. 2017 Aug 3;8(48):83370–83. doi: 10.18632/oncotarget.19848 (PMC5663522; doi:10.18632/oncotarget.19848)
Supplement: Supplementary file 1 [file oncotarget-08-83370-s001.pdf]

IL2RG, identified as overexpressed by RNA-seq profiling of pancreatic intraepithelial neoplasia, mediates pancreatic cancer growth

SUPPLEMENTARY MATERIALS

Alternative transcription analysis

We compared the transcriptional profiles of PanIN3 and normal pancreatic duct samples. We generated a list of 3053 exon inclusion events mapping to known genes. We did not observe a reduction in splicing diversity that had been reported in pancreatic cancer cell lines [3]. 1200 of the 3053 events were predicted to result in underexpression of the corresponding isoform in PanIN-3 samples and 1853 transcripts were predicted to result in an overexpression.

Many of the alternatively spliced transcripts were of unknown biological significance, but a few stood out based on literature review and were selected for additional analysis. The MUC1 gene possesses a variable number of tandem repeats (VNTR) region and has 20 amino acids subject to variable O-glycosylation. In over 80% of pancreatic adenocarcinomas MUC1 is differentially glycosylated which can affect the chemosensitivity of cancer [8]. In our list of candidate alternatively spliced transcripts, two of the highest scored transcripts (-0.82345, adjusted p-value = 0.00013;-0.74755, adjusted p-value = 0.00117) occur in MUC1. These splicing events map to an isoform previously described as oncogenic [4]. Annexin 2 (ANXA2) has several alternative splicing events, including one highly-scoring for inclusion in PanIN3 samples over normal pancreatic duct samples (-0.63655) that suggests a switch between two known isoforms. The functional significance of these isoforms is unknown, but ANXA2 is being investigated as a target for immunotherapy [10].

Three of the highest-scoring events (-0.75227, -0.72576, -0.72576) were for Myosin X (MYO10) transcripts, an unconventional myosin that acts to form filopodia on the cell surface (Berg, 2002). MYO10 has recently been established as playing an important role in cancer invasion and metastasis through construction of invadopedia, cell protrusions that are capable of penetrating and digesting extracellular matrix (Schoumacher 2010). MYO10 knockdown results in a considerable reduction of invadopedia formation and matrix digestion [2]. MYO10 overexpression promotes aggressiveness and metastasis in breast cancer, and its expression is induced in pancreatic cancer by the introduction of mutant p53 in mouse models [1, 2]. Silencing of MYO10 in breast and PDAC cells inhibits invasion [1].

MYO10 consists of two functionally-distinct isoforms, "full" (fMYO10) and "headless" (hMYO10). fMYO10 is connected to mitosis and cell migration in neurons, while hMYO10 is a dominant negative inhibitor of full[6]. The three splicing events reported correspond to inclusion of exons 18 and 19, which are part of the fMYO10 isoform. Counts of this inclusion event indicate that PanIN3s undergo a drastic alternative splicing event from a full: headless ratio of 7 : 42 in our normal pancreatic duct samples to 56 : 7 in our PanIN3 samples. Notably, in our differential expression comparison, MYO10 fell short of the threshold for significance with an upregulation of 2.07-fold over the normal duct samples (see Supplemental Figure 1). Multiplying this fold-ratio by the calculated isoform ratio, PanIN3 samples upregulate fMYO10 by 16.57-fold over the normal pancreatic ducts and downregulate hMYO10 by 3-fold.

RT-PCR with isoform-specific primers for MUC1, ANXA2, and MYO10 found that the spliced isoforms overexpressed in PanINs were also overexpressed in pancreatic cancer cell lines Panc5.04, MiaPaCa2, and Panc8.13 in comparison with the non-neoplastic pancreatic duct line HPDE (Supplemental Figure 1). Both isoforms of MUC1 had comparable expression in pancreatic cancer cell lines, while for ANXA2 the normal isoform was more overexpressed than the PanIN isoform. The full isoform MYO10, putatively oncogenic, was overexpressed than the headless isoform in pancreatic cancer cell lines in comparison to HPDE.

Fusion transcripts in PanINs

Sixteen candidate fusion transcripts were identified each of which was only detected in one PanIN sample (see Supplemental Table S5). For six of these events, one or both fusion partners has been previously reported in human cancer, although none of them have been reported in a pancreatic cancer [7].

These partners include: HINT1, TJP1, DNM2, LARGE, MLLT10, PP2R1B, and FTFT1. A few of the partners have promising roles. The region of DNM2 included in one fusion contains miRNA199-a, which is oncogenic in pancreatic cancer

[9]. HINT1 has been described as having tumor suppressor functions in certain contexts. We detect a HINT1-NECAP2 fusion in which HINT1 has a frameshift of 2 base pairs, possibly resulting in its inactivation. NECAP2 has been recently tied to EGFR endocytosis in breast cancer [5].

Supplemental Methods

RT-PCR

To examine levels of alternatively spliced transcripts in PDAC, RNA was harvested from pancreatic cancer cell lines and pancreatic cancer patient tumor samples. 1µg total RNA from each sample was reverse transcribed using QuantiTect Reverse Transcription Kit (Qiagen) by the manufacturer's protocol. The resulting cDNA was diluted 1:200 on the ABI 7300 Real-Time PCR thermocycler (Applied Biosystems) using SYBR Green PCR Master Mix and recommended PCR conditions (Applied Biosystems). Melting curve analysis and normalization to 18S rRNA was performed.

Primers were designed to target isoform-specific regions of MUC1, ANXA2, and MYO10 and ordered from Integrated DNA Technologies (www.idtdna.com):

ANXA2 Normal Isoform F1 5'- CCAGGGTGAAAATGTTTGCCA
ANXA2 Normal Isoform R1 5'- GCTTGCACAGGATTTCGTGA
ANXA2 PanIN3 Isoform F1 5'- GGCTGCCCACTTCCTTCAAA
ANXA2 PanIN3 Isoform R1 5'- TCCCGCTCAGCATCAAAGTT
MUC1 Normal Isoform Exon5-6 F1 5'- AGCGTGAGTGATGTGCCATT
MUC1 PanIN3 Isoform Exon5-6 F1 5'- TCAGCGGCTGTCTGTCAGT
MUC1 Isoform-Agnostic Exon5-6 R1 5'- CGCCCATGGGTGTGGTAG
MYO10 Full Isoform F1 5'- GATAACTTCTTCACCGAGGGAACA
MYO10 Full Isoform R1 5'- CCGGCTGTACTGCTCCATGGTGGCAG
MYO10 Headless Isoform F1 5'- CAGCACAGCCCGAGACGCAC
MYO10 Headless Isoform R1 5'- CTTGGGTTTTCTTTCACCCCTTCAG

REFERENCES

1 Arjonen A, Kaukonen R, Mattila E, Rouhi P, Hognas G, Sihto H et al. Mutant p53-associated myosin-X upregulation promotes breast cancer invasion and metastasis. J Clin Invest 2014; 124: 1069-1082.

2 Cao R, Chen J, Zhang X, Zhai Y, Qing X, Xing W et al. Elevated expression of myosin X in tumours contributes to breast cancer aggressiveness and metastasis. British journal of cancer 2014; 111: 539-550.

3 Carrigan PE, Bingham JL, Srinvasan S, Brentnall TA, Miller LJ. Characterization of alternative spliceoforms and the RNA splicing machinery in pancreatic cancer. Pancreas 2011; 40: 281-288.

4 Chaika NV, Gebregiworgis T, Lewallen ME, Purohit V, Radhakrishnan P, Liu X et al. MUC1 mucin stabilizes and activates hypoxia-inducible factor 1 alpha to regulate metabolism in pancreatic cancer. Proc Natl Acad Sci U S A 2012; 109: 13787-13792.

5 de Graauw M, Cao L, Winkel L, van Miltenburg MH, le Devedec SE, Klop M et al. Annexin A2 depletion delays EGFR endocytic trafficking via cofilin activation and enhances EGFR signaling and metastasis formation. Oncogene 2014; 33: 2610-2619.

6 Ju XD, Guo Y, Wang NN, Huang Y, Lai MM, Zhai YH et al. Both Myosin-10 isoforms are required for radial neuronal migration in the developing cerebral cortex. Cereb Cortex 2014; 24: 1259-1268.

7 Klijn C, Durinck S, Stawiski EW, Haverty PM, Jiang Z, Liu H et al. A comprehensive transcriptional portrait of human cancer cell lines. Nat Biotechnol 2015; 33: 306-312.

8 Nath S, Daneshvar K, Roy LD, Grover P, Kidiyoor A, Mosley L et al. MUC1 induces drug resistance in pancreatic cancer cells via upregulation of multidrug resistance genes. Oncogenesis 2013; 2: e51.

9 Shatseva T, Lee DY, Deng Z, Yang BB. MicroRNA miR-199a-3p regulates cell proliferation and survival by targeting caveolin-2. J Cell Sci 2011; 124: 2826-2836.

10 Zheng L, Jaffee EM. Annexin A2 is a new antigenic target for pancreatic cancer immunotherapy. Oncoimmunology 2012; 1: 112-114.

For Supplementary Tables see in Supplementar Files.
